# HIFU Drive System Miniaturization Using Harmonic Reduced Pulsewidth Modulation

**DOI:** 10.1109/TUFFC.2018.2878464

**Published:** 2018-10-29

**Authors:** Chris Adams, Thomas M. Carpenter, David Cowell, Steven Freear, James R. McLaughlan

**Affiliations:** School of Electronic and Electrical EngineeringUniversity of Leeds 4468 LeedsLS2 9JTU.K.

**Keywords:** Ablation, cavitation, electronics, high-intensity focused ultrasound (HIFU), phased array, system design

## Abstract

Switched excitation has the potential to improve on the cost, efficiency, and size of the linear amplifier circuitry currently used in high-intensity focused ultrasound (HIFU) systems. Existing switching schemes are impaired by high harmonic distortion or lack array apodisation capability, so require adjustable supplies and/or large power filters to be useful. A multilevel pulsewidth modulation (PWM) topology could address both of these issues but the switching-speed limitations of transistors mean that there are a limited number of pulses available in each waveform cycle. In this study, harmonic reduction PWM (HRPWM) is proposed as an algorithmic solution to the design of switched waveforms. Its appropriateness for HIFU was assessed by design of a high power five-level unfiltered amplifier and subsequent thermal-only lesioning of *ex vivo* chicken breast. Three switched waveforms of different electrical powers (16, 26, 35 W) were generated using the HRPWM algorithm. Lesion sizes were measured and compared with those made at the same electrical power using a linear amplifier and bi-level excitation. HRPWM produced symmetric, thermal-only lesions that were the same size as their linear amplifier equivalents ($p > 0.05$). At 16 W, bi-level excitation produced smaller lesions but at higher power levels large transients in the acoustic waveform nucleated undesired cavitation. These results demonstrate that HRPWM can minimize HIFU drive circuity size without the need for filters to remove harmonics or adjustable power supplies to achieve array apodisation.

## I. Introduction

High-intensity focused ultrasound (HIFU) is a noninvasive surgical technique that is used to generate coagulative necrosis in tissue through localized thermal ablation [1], [2] and other mechanical effects [3], [4].

The main application areas of HIFU are the treatment of soft tissue tumors [5], [6] in liver [7], kidney [8], prostate [9], [10], breast [11], and in the brain [12], [13]. HIFU is not limited to the treatment of soft tissue tumors, and exploration of new avenues such as triggered drug delivery [14], treatment of bone tumors [15], neurological disorders [16], ectopic implantation [17], and pain management [18], [19] continues.

Single-element transducers with a fixed focus have traditionally been used to achieve the desired intensities for ablation [20]. Recently though, high-power therapeutic arrays are increasingly used [21] as they can facilitate dynamic focal position for hyperthermia [22], [23] and ablation [24]. Large arrays on the order of 1000 elements are essential in transcranial therapy to spread the acoustic heating in the skull [25], [26]. Since the attenuation and phase aberrations induced by the skull vary considerably across its surface [27], [28], the phase and amplitude of the excitation waveform of each element must be adjusted [29]–[31]. Other uses of array transducers include rib sparing in the treatment of liver [32]–[34], volumetric treatment of uterine fibroids [35], and surgery on the prostate [36]. While the ability to excite each element in the array with a different waveform is essential to transcranial therapy, it is also highly desirable in other applications as it facilitates array apodisation which reduces side lobes, linear frequency modulation to reduce grating lobe energy [37], phase shift keying [38] to suppress standing waves, and beam steering [39].

In HIFU systems, each array element is typically connected to a linear power amplifier so that the system can deliver the high electrical powers (> 15 W) necessary for tissue ablation. Each amplifier is in turn connected to its own waveform generator to achieve the necessary phase and amplitude control. This arrangement has low harmonic distortion, meaning that the electrical waveform does not contain undesired harmonics of the fundamental component. While this is desirable, there are a number of disadvantages including high cost, low efficiency, and large size [40]. This is increasingly problematic as higher density arrays continue to be developed. In addition, the large numbers of passive components which are prevalent in these designs limit their usefulness for catheters and in MRI environments [41]. For HIFU array treatments to be more financially and practically accessible, improvements should be made to the driving circuitry [40].

In this paper, the harmonic reduction pulsewidth modulation (HRPWM) algorithm is proposed to reduce the size and cost of the excitation circuitry currently used in HIFU array systems. Comparisons will be made with existing excitation techniques numerically and experimentally.

## II. Switching Schemes and Amplifier Design

Switched mode circuits have numerous advantages over linear amplifier designs [41], [42]. Unlike linear amplifiers, the input of each transducer element is connected to transistors that rapidly switch between a discrete number of voltage levels to approximate the desired waveform. Operating the transistors in their saturated region increases the efficiency but may produce powerful third and fifth harmonics in the electrical waveform [43]. For example, when a simple bi-level excitation is used, the third and fifth harmonics are only 10 and 15 dB less powerful than the fundamental component. These harmonics can be within the bandwidth of HIFU transducers which are highly resonant devices that produce acoustic energy at their harmonics [44], [45]. These harmonics could cause unwanted effects [46], such as disruption of the focal region [41], heating of the transducer [47], and the generation of grating lobes [48].

### A. Switching Schemes

A number of switching schemes have been proposed as an alternative to linear amplifiers. Bi-level has been shown to minimize the footprint of driving electronics and improve efficiency. Amplitude control can also be achieved by adjusting the duty cycle [49], but if the switching frequency is within the bandwidth of the transducer, this regime still requires additional fixed-frequency filtering components in the circuit design to remove harmonics. These filters are large, MRI incompatible [50], and limit the use of frequency modulated waveforms.

Tang and Clement [43] demonstrated that introducing a predetermined off period between the two levels can be used to disrupt the periodicity of the third harmonic. This has been implemented in an MRI compatible catheter system [51]. Alternatively, additional levels can be introduced to the circuit to implement staircase converters. This naturally disrupts the generation of harmonics [52] and several authors have demonstrated harmonic reduction this way at ever increasing frequencies and powers [47], [53]. However, the constraints placed on the waveform with these techniques mean that each array element would require its own adjustable supply to achieve apodisation which is costly and cumbersome.

Amplitude control in a switched system can be achieved using PWM and has been successfully applied to ultrasonic imaging [54]. Here, the circuitry is continually switched at a higher rate than the frequency response of its load. The load acts as a bandpass filter causing averaging of the drive voltage across one switching period. The pulsewidth is then modulated to achieve the desired instantaneous amplitude. It is possible to drive a HIFU transducer with PWM, but the combination of high power and frequency, mean that a limited number of pulses per waveform are available, and thus the operator may have limited control over the amplitude.

HRPWM is a five-level PWM scheme with carriers algorithmically designed specifically to reduce the number of pulses in each cycle while retaining amplitude control and actively implementing harmonic reduction [55]. It takes a number of inputs including frequency, bandwidth (for chirps), and sampling frequency and produces a five-level waveform for any suitable switched circuit or pulser. It has facilitated a number of medical imaging and nondestructive testing applications [56]–[58], but this study is the first time it has been used for HIFU.

### B. Circuit Operation

Previous publications on HRPWM have utilized five-level integrated pulser ICs to excite the transducer. These integrated devices have limited total continuous power ratings, so are not suitable for the continuous wave (CW) operation necessary for HIFU. For this study, a purpose built five-level pulser bridge circuit was designed using discrete components to facilitate high-power CW operation.

**Fig. 1.**
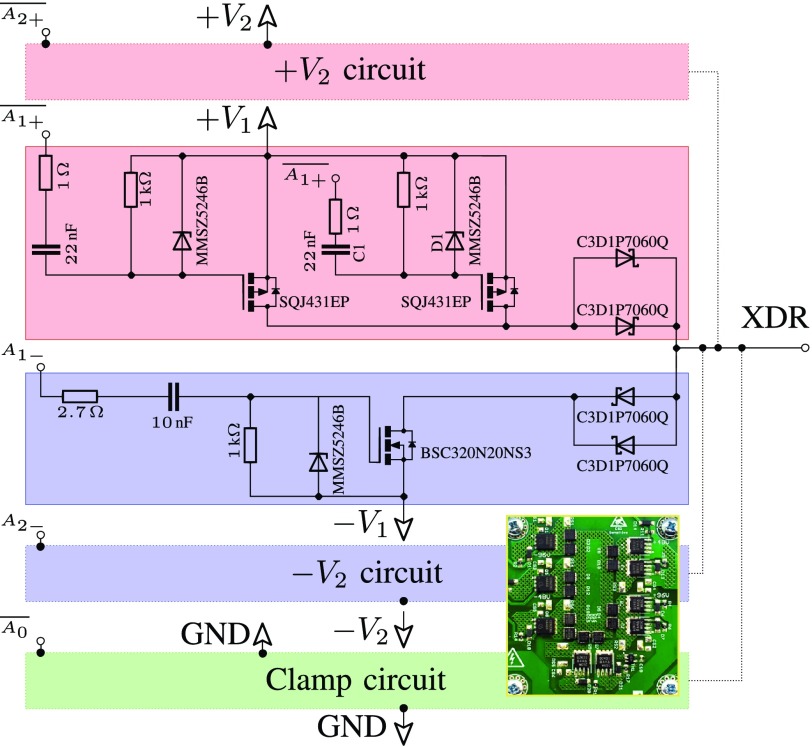
Part of the circuit design used in this study [59]. The red areas represent components used for positive levels, while blue represents areas used for negative and green for ground (clamp). The circuit output is connected to the transducer element without filters. Inset: active area of a fabricated card which consumes approximately 25 cm^2^ of space.

Fig. 1 shows the structure of one bridge of the pulser. Each pulser is made up of three identical bridges, providing $\pm {V_{1}}$, $\pm {V_{2}}$, and ground rails. The full schematic and printed circuit board (PCB) design are available online [59]. Each bridge consists of two capacitive-level shifter circuits, a nMOS power transistor for the negative rail, a pair of pMOS transistors for the positive rail, and two pairs of diodes to prevent the MOSFET body diodes becoming forward biased. Two pMOS transistors are used in parallel as the series resistance of each pMOS device is about twice that of the nMOS transistors. The pulser is controlled by driving the six MOSFET gates, two per bridge. The pMOS transistors are controlled by the active low signals $\overline {A_{0}}$, $\overline {A_{1+}}$, and $\overline {A_{2+}}$ where a 0 V pulse will turn the MOSFET ON, and +12 V will turn it OFF. For each nMOS transistor, a +12 V pulse on the active high control signals $A_{0}$, $A_{1-}$, and $A_{2-}$ will turn it ON and 0 V will switch it OFF. These control signals require relatively high switching currents due to the large capacitance of the MOSFET gates, and as such are driven using MOSFET drivers (Analog Devices ADP3654) with current limiting resistors. As the gates are referenced to high-voltage power supplies ($V_{n} \approx 30 {\mathrm { \text {V}}}$), capacitive-level shifters are used to isolate the gates from the control circuitry. The level shifters use series capacitors that are biased to the supply voltages via a combination of a parallel resistor and Zener diode. When the high-voltage power supplies are turned ON, the capacitors will rapidly charge up to the supply voltage through the Zener diode, which protects the gates. When the input control signal side of the capacitor is driven to “ON” (+12 V for nMOS, 0 V for pMOS), this change in voltage will be reflected as an equal change at the gate, becoming $-V_{n}+12 {\mathrm { \text {V}}}$ and $+V_{n}-12 {\mathrm { \text {V}}}$ for nMOS and pMOS, respectively. Similarly, when the control signal is driven to “OFF”, the gate side of the capacitors will return to the supply voltage $\pm V_{n}$. Due to the bleed resistor in the level shifter, the $\overline {A_{1+}}$, $\overline {A_{2+}}$, $A_{1-}$, and $A_{2-}$ control signals cannot be driven to “ON” indefinitely, being limited to approximately 1 ms on-time. This is not an issue for ultrasound applications as each gate is typically turned ON for less than a microsecond. The pulser circuit was fabricated on a PCB with an active area of approximately 25 cm^2^ as shown in Fig. 1 (inset). 16 channels of the drive circuit were connected to a field-programmable gate array (FPGA) development card (5SGSMD5N, Altera, USA). The transmit waveforms were then designed using MATLAB (Mathworks, USA) and uploaded via peripheral component interface (PCI) express to the FPGA. Each channel could be operated individually with up to 41 W output power for 30 s of CW, or combined in parallel for higher output powers.

## III. Numerical Study: Effect of Harmonic Distortion on Lesioning

Numerical simulations were undertaken to assess the effect of harmonic distortion from switched excitation on lesion formation. The simulation considered a single-element transducer where amplitude control can be achieved by adjusting the power supply. This allows the effects of harmonic distortion to be isolated from any amplitude control capabilities that are necessary in array systems.

Two-dimensional simulations were performed in the pseudospectral simulation package k-wave [60]. A typical concave HIFU transducer with a diameter of 64 mm, a natural focus of 63 mm, and a center frequency of 1.1 MHz was simulated. The transducer transfer function consisted of a low pass, 50-order finite-impulse response (FIR) filter that rejected energy at harmonic four and above. The simulation consisted of $571\times 696$ elements of which each represented $\mathrm {112~ \mu \text {m} }$, leading to a total useful area of $64 {\mathrm { \text {m} \text {m} }}\times 78 {\mathrm { \text {m} \text {m} }}$. A 20 element-thick absorbing boundary was placed around the perimeter to stop reflections. The transducer was oriented so that the propagation direction was the same as the longest side in the simulation area.

**Fig. 2.**
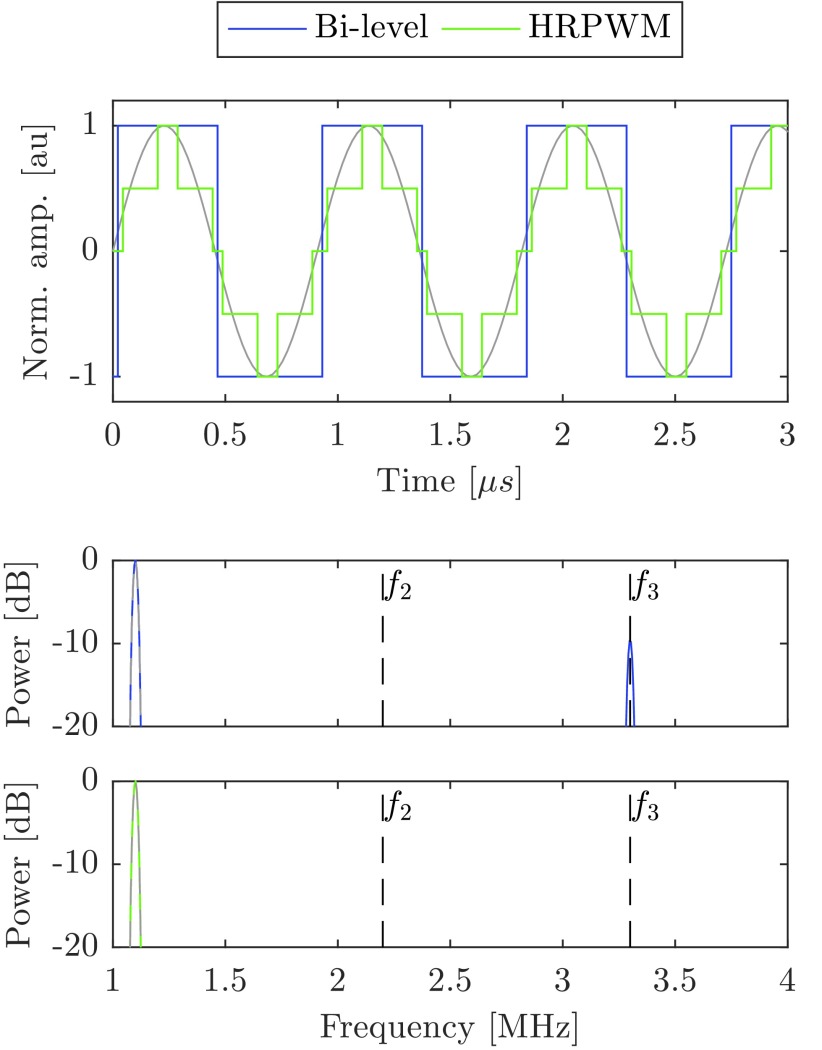
Time and frequency domain plots of the 1.1 MHz bi-level and HRPWM excitations used in simulation. Ideal linear amplifier results are shown in gray for comparison. The second and third harmonics of the transducer are indicated by the black lines $f_{2}$ and $f_{3}$.

Bi-level and HRPWM excitation schemes were considered in conjunction with a linear amplifier. A 50% duty cycle square wave was used for the simplest of the switched schemes, bi-level excitation. For HRPWM, transitions to each level are set to achieve the desired magnitude of the fundamental component while ensuring that any switching-induced harmonics destructively interfere. Pertinent to the algorithm is that its carriers can change in frequency and phase, allowing the waveforms to be designed to give only one or two pulses per half cycle. This ensures robust control of amplitude and thus acoustic intensity using a minimal number of switching events. The acoustic wattage produced by the transducer was maintained between schemes. This allowed the effects of harmonic distortion in the presence of tissue to be assessed. To this end, the virtual power supply of each of the schemes was adjusted so that when the transducer transfer function was applied they all produced 26 W of acoustic power. In addition to control by supply rails, the HRPWM scheme is also able to modulate its own amplitude so it was arbitrarily set to produce waveforms at 70% duty cycle prior to adjustment of the power supply. Fig. 2 shows the bi-level and HRPWM schemes in the frequency and time domains. The two example excitations are compared with a perfect sinusoidal excitation by a linear amplifier which is shown in gray. Three cycles of the excitations are shown. In the frequency domain, the highest 20 dB of normalized amplitude is shown across the frequency response of the transducer. The location of the second and third harmonics of the transducer, where the electrical conversion efficiency is highest, is indicated by the lines $f_{2}$ and $f_{3}$. Below 20 dB, any energy is considered irrelevant. Neither the sinusoidal nor HRPWM excitation has observable frequency content outside the fundamental component. The bi-level scheme has harmonic distortion at $f_{3}$ that could be converted into acoustic energy. The transducer was placed in water and a 45 mm-thick medium that best represented chicken muscle [61], [62] was placed at the transducer’s focal point. The medium’s acoustic and thermal parameters are presented in Table I.

**TABLE I:** Medium Properties Used for Modeling

Property	Symbol	Value
Speed of sound	$c$	$\mathrm {1520~ \text {m} \, \text {s}}^{-1}$
Density	$\rho $	$1020 ~\text{kg}\, \text{m} ^{-3}$
Thermal conductivity	$\Lambda $	$\mathrm {0.5~ \text {W} \,\text {m}^{-1} \text {K} }$
Specific heat	$C$	$\mathrm {3600~ \text {J} \,\text {kg}^{-1} \text {K} }$
Ambient temperature	$T_{0}$	37 °C
Attenuation	$\alpha $	$\mathrm {1.1~ \text {dB} / \text {M} \text {Hz} ^{1.5} \text {c} \text {m} }$
Parameter of nonlinearity	$(B/A)$	6.4

Nyborg’s heating equation [63] was modified to calculate the total heat generation by the ultrasound at the first six harmonics

\begin{equation*} Q = \sum _{h=1}^{h=6}\alpha _{h} p_{h}^{2}\big /\rho c\tag{1}\end{equation*}

where $p_{h}$ and $\alpha _{h}$ refer to the magnitude of the pressure and absorption at harmonic $h$. Pennes’ bioheat transfer equation [64] was then used to calculate the temperature rise in the medium for each exposure made with each scheme. Exposures were made for 20 s before cooling for a further 10 s, to account for perfusion. The starting temperature was 37 °C. From the thermal exposures, CEM43 was calculated [65]. A lesion map was then created from the area of tissue where CEM43 exceeded 240 min, which is the recommended value for the simulation package and also the reported damage threshold for prostate tissue [66].

## IV. Experimental Study: Lesion Volume Control With PWM

A key advantage of the HRPWM scheme is the ability to apodise arrays without the need for independent adjustable power supplies or external filters. It is essential that this apodisation can occur at electrical powers relevant for HIFU therapy. In this section of the study, assessment of the scheme’s amplitude control capability was made by comparing lesioning efficacy with a linear amplifier. A reduction in amplitude should reduce the lesion volume and vice versa. Additional comparisons were made with bi-level excitation. The aims of the experimental study were to both augment the simulation results and to assess the capability of HRPWM to control acoustic intensity and duration at lesioning levels.

**Fig. 3.**
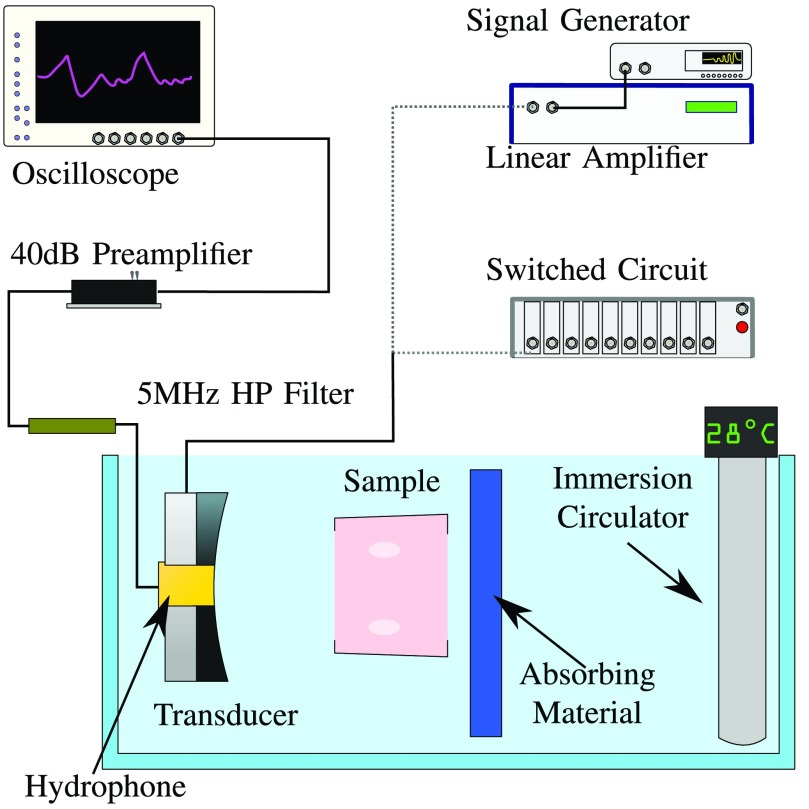
Schematic of the experimental apparatus used in this study. Not shown: current probe, matching network, and CNC machine.

For the linear amplifier experiments, a signal generator (33600A, Agilent, USA) was connected to 45 dB linear power amplifier (A150, E&I Ltd, USA) (Fig. 3).

### A. Sample Preparation, Lesioning, and Analysis

Ex vivo chicken breast was used for the lesioning study. Fresh chicken breasts were lesioned within 18 h of purchase and were refrigerated at 4 °C when not used. The tissue was cut into cubes approximately 55 mm $\times \,\,55$ mm $\times \,\,40$ mm. The samples were then degassed in a 1% (v/v) phosphate buffer solution for 4 h. To ensure repeatability between samples, they were placed in a holder marginally smaller than their cut size so that they were slightly compressed in all directions. The sample holder had acoustic windows on opposite sides of approximately 50 mm $\times \,\,50$ mm (Fig. 3).

Lesioning was performed with a single-element focused HIFU transducer (H-102, Sonic Concepts, USA) in conjunction with the manufacture provided impedance matching network, although it contained no purpose frequency filtering components. The transducer had a center frequency of 1.1 MHz, a focal distance of approximately 63 mm, and a diameter of 64 mm. The −6 dB beamwidth was 1.33 mm in diameter and 10 mm long. To colocate the centers of the transducer focus and the samples, an alignment target was temporarily attached to the inside of the sample holder, prior to the start of the first exposure. Using a hydrophone (Y-107, Sonic Concepts, USA) colocated and confocal with the center of the HIFU transducer, the transducer focus was pulse echo positioned onto the target. The sample holder was attached to a computer numerically controlled (CNC) machine stage which was programed to move to five fixed locations spaced 20 mm apart. This meant that the five lesions were always made in the same places and at a fixed depth of 20 mm in each sample. Sonications were performed in a tank of degassed, deionized water which was maintained at 28 ± 1 °C using an immersion circulator. This temperature was chosen to be representative of *in vivo* tissue without causing premature denaturing of the sample.

To attenuate postfocal energy and prevent reflections, 10 mm of absorbing material was placed behind the samples. This arrangement is depicted in Fig. 3. The samples were sonicated for 20 s. Between exposures, the tissue was allowed to cool for 10 s, to allow the bulk temperature of the tissue to return to ambient. Immediately after all the exposures were complete, samples were sliced through the center of the lesions, revealing two halves of each lesion. Photographs of each lesion were taken next to a ruler and an identifying code. Using image analysis software (ImageJ, National Institutes for Health, Bethesda, MD, USA), the pixel/size ratio was calculated and then the lesion cross-section area was measured using the ellipse area tool.

For the lesioning efficacy of different excitation schemes to be compared, we aimed to ensure that damage to the tissue was predominantly thermal in nature and not from mechanical effects such as acoustic cavitation and/or boiling [67]. Three measures were taken to ensure that mechanical damage was reduced. First, a passive cavitation detection (PCD) system was used [67]. Second, exposure times and intensities within the limits of previously published lesioning work (in chicken breast) were used [68]. Third, lesions were inspected for unusual shapes that may suggest boiling.

For the PCD system, the hydrophone was connected to an 11-bit oscilloscope (MSO-5104A, Agilent, USA) via a 5 MHz high-pass filter (THP5P554100B, Allen Avionics, Mineola, NY, USA) and a 40 dB preamp (SPA1411, Spectrum, Germany). The high-pass filter was used to remove any reflected energy from the HIFU transducer’s first three harmonics and to avoid saturation of the oscilloscope’s input. The oscilloscope was run in segmented mode, and was set to record up to 256 waveforms of 250$\mu \text{s}$ length. The trigger threshold was set slightly above the noise level at 380 mV. The trigger hold-off was set to 50 ms so that the data from up to 12.8 s of the exposure could be recorded. The number of triggers was recorded for each exposure, and if this number exceeded 10, the lesion was considered to be mechanically rather than thermally formed. Fourier analysis was performed on the recorded signals.

To ensure that each tissue sample was adequately degassed, prior to lesion formation, a high amplitude ($\approx 2$ MPa) 5-cycle pulse was applied to the transducer to discount the presence of bubbles. It was expected that the presence of bubbles would have a harmonic response, and therefore, trigger the PCD. In addition, every tissue sample contained at least one thermal-only lesion made using the linear amplifier. This meant that if cavitation was observed the exposure parameters could be isolated as the cause of cavitation.

### B. Considered Schemes and Control of Acoustic Intensity

HRPWM, linear amplifier, and bi-level schemes were used. Three different electrical powers for each scheme were considered: 16, 26, and 35 W. Using the beamwidth from the transducer datasheet and presuming an 80% efficiency, these electrical powers correspond to intensities of 1205, 1958, and 2637 $\mathrm { \text {W} / \text {c} \text {m} ^{-2}}$, respectively. Prior to ablations, with the transducer *in situ* but samples removed, the circuit was adjusted to achieve the desired electrical power. For the linear amplifier experiments, the signal generator amplitude was adjusted. For the bi-level excitation, the supply voltage was adjusted but for HRPWM, the power supplies were fixed and the amplitude modulation parameter was changed.

If knowledge of a transducer’s complex impedance is known and a single-drive frequency is used to excite the transducer, delivered true power can be controlled by changing the voltage of the waveform [69]. This approach is unsuitable here for two reasons: 1) the switched waveforms contain multiple frequency components that have differing corresponding impedances and 2) the output impedance of the switched circuitry is unknown and so there is potential for high levels of reflected energy.

Instead, a current probe (TM502A, Tektronix Inc., USA) and an oscilloscope (DS06014A, Agilent, USA) were used to measure the total delivered true power. The current probe was placed around the positive input of the matching network. Using a 100-cycle excitation, true power as a function of frequency was then calculated from the real product of the Fourier transformed voltage and conjugated current waveforms

\begin{equation*} P(f) = \text {Re}(V(f) \times I(f)^{*}).\tag{2}\end{equation*}

The total delivered power across a given frequency range was then calculated from the integral of $P(f)$

\begin{equation*} P_{T} = \int _{f_{0}}^{f_{1}} P(f) df.\tag{3}\end{equation*}

For the experiments, 500 KHz and 10 MHz were used for $f_{0}$ and $f_{1}$, respectively.

Since the electrical and acoustic configuration was consistent between schemes, it was presumed that the acoustic intensity remained the same irrespective of the scheme used. Before the transducer was connected to obtain the parameters used for the study, this power measurement approach was successfully tested using a purely resistive 50 $\Omega $ load.

For each intensity and scheme, three lesions were made and measured, leading to a total of 27 exposures.

## V. Results and Discussion

**Fig. 4.**
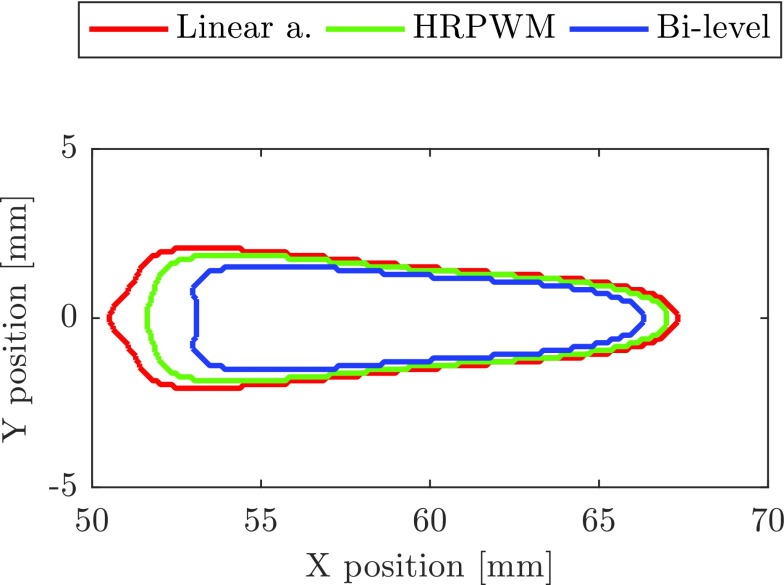
Simulated lesioning results. HRPWM and bi-level switched schemes were compared with excitation by a linear amplifier.

Fig. 4 shows the simulated lesioning formation due to ultrasound exposure under the three different excitation schemes. The acoustic field would propagate from left to right. The lesions are correctly shaped and axisymmetric although not rotationally symmetric. This is because of prefocal lesioning due to nonlinear propagation [20], [70]. The lesion cross-section areas and their percentage changes from the linear amplifier lesion are given in Table II.

**TABLE II:** Comparison of Simulated Lesion Sizes With Different Excitation Schemes

Excitation	Lesion Cross Section Area (mm^2^)
Linear Amplifier (Ideal)	49
HRPWM	43 (−12%)
Bi-level	32 (−35%)

Ideally, each of the simulated schemes would have been calibrated to produce the same acoustic intensity at the focal region. However, this information cannot be obtained, prior to completion of a free-field simulation. Haar *et al.* [71] have suggested previously that peak acoustic pressures might instead be appropriate to make comparisons between lesioning experiments. However, this does not consider the increased energy that exists in the acoustic waveform when harmonics are introduced. For this reason, each scheme was calibrated using a fixed acoustic wattage at the surface. Despite this calibration, the schemes all produced different lesion sizes in the simulation. When bi-level excitation was used, the lesions were smaller (−35%) compared with linear amplifier excitation. This is because more of the acoustic energy was distributed in harmonics and as the higher frequency components were more readily absorbed, less energy reached the focal region. When HRPWM excitation was used, the lesion cross-section area was only slightly smaller (−12%) than the linear amplifier lesion. This is likely due to HRPWM having energy at higher order harmonics that are attenuated by the tissue.

The simulation results show that an increase in harmonic distortion of the excitation waveform reduces the lesion size (Fig. 4). The HRPWM scheme had the lowest harmonic distortion of the switched schemes and thus produced a lesion that was much closer in size.

**Fig. 5.**
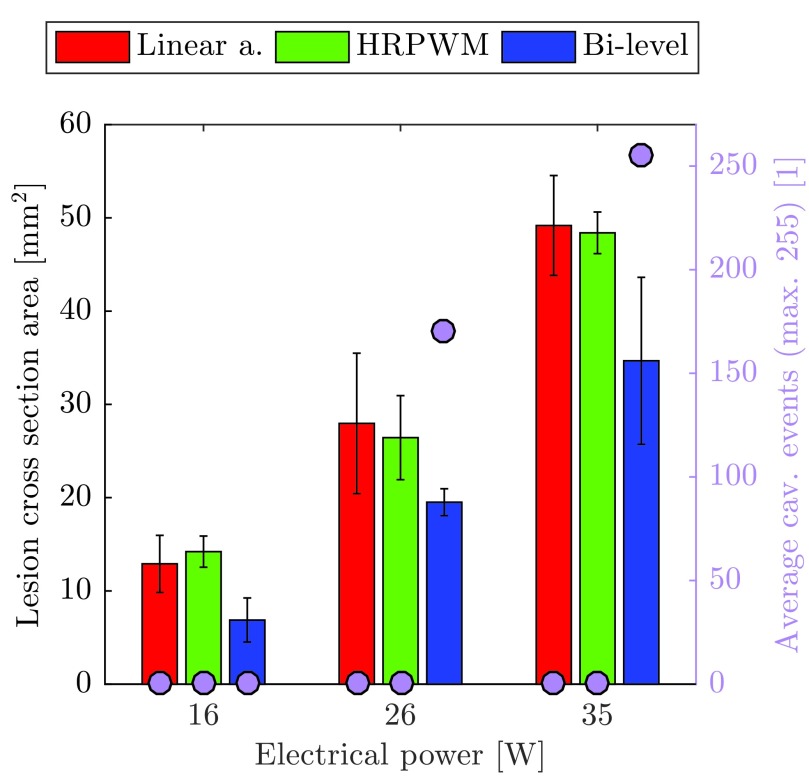
Lesion cross-section areas at several electrical powers using different excitation schemes. Error bars are calculated from the standard deviation of three repeats at each scheme and power. Purple markers: number of recorded cavitation events on average for each of the excitations.

Fig. 5 shows the averaged lesion cross-section areas in *ex vivo* chicken breast, for different electrical powers and excitation schemes. The error bars represent the standard deviation of the three measurements, where each measurement corresponds to a new lesion formed and measured by the same operator. The variance in mean lesion area between the linear amplifier and HRPWM was between +10% at 16 W, and −5% at 26 W. For bi-level, the variance was between −30% at 26 W and −45% at 16 W.

Each set of repeats were tested for normality using the Shapiro–Wilk test which is ideal for small sample sizes [72]. All sets of repeats did not reject the null hypothesis ($p_{\mathrm {\mathrm {sw}}} > 0.05$) except for the lesions produced using 35 W bi-level excitation ($p_{\mathrm {\mathrm {sw}}} < 0.05$). Analysis of variance (ANOVA) was then undertaken on the lesion cross-section areas to quantify their statistical significance. The tests and results are summarized in Table III. ANOVA was performed to assess two attributes: 1) similarity between schemes at the same power (rows 2–4, $p_{\mathrm {av}2}$) and 2) difference in lesion volume at different powers with fixed schemes (row 1, $p_{\mathrm {av}1}$). The analysis shows that a change in electrical power yields a change in lesion size ($p_{\mathrm {av}1} < 0.05$) with all schemes. With HRPWM and linear amplifier, the sizes are similar at each power level ($p_{\mathrm {av}2} > 0.05$).

**TABLE III:** ANOVA Tests

Wattage (W)	Test	Value
16–35	Linear Amplifier	$p_{\mathrm {av}1}=0.0006$
	Bipolar	$p_{\mathrm {av}1}=0.0023$
	HRPWM	$p_{\mathrm {av}1}< 0.0001$
16	Lin. vs Bipolar	$p_{\mathrm {av}2}=0.0543$
	Lin. vs HRPWM	$p_{\mathrm {av}2}=0.5517$
26	Lin. vs Bipolar	$p_{\mathrm {av}2}=0.1290$
	Lin. vs HRPWM	$p_{\mathrm {av}2}=0.7788$
35	Lin. vs Bipolar	$p_{\mathrm {av}2}=0.0737$
	Lin. vs HRPWM	$p_{\mathrm {av}2}=0.8264$

With 16 W bi-level, the ANOVA suggests that even though the mean lesion size ($\mu $) is 45% lower than when using the linear amplifier, the results are statistically insignificant ($p_{\mathrm {av}1} > 0.05$). This is likely a false positive due to the sample size being small. Compared with other schemes, the bi-level results are much less discriminated between powers (0.0023 > 0.0001). At the two higher power levels, there is better agreement with HRPWM and linear amplifier than with bi-level and the linear amplifier excitation ($0.8264 \gg 0.0737$ at 35 W).

The standard deviation ($\sigma $) for the 26 W bi-level lesions is smaller than with other excitations. However, no significance is attached to this, as the coefficient of variance ($\sigma /\mu =0.0740$) is similar to values observed elsewhere, e.g., HRPWM 35 W ($\sigma /\mu =0.0462$). The authors believe, therefore, that the contributors to the standard deviation were mainly variations in tissue and experimental error.

**Fig. 6.**
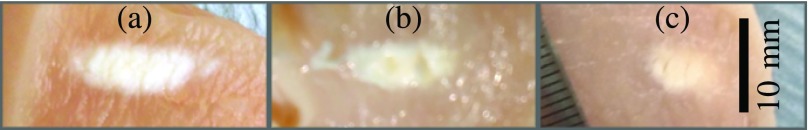
Lesions made using 35 W of electrical power. (a) Lesion was made using the linear amplifier. (b) Lesion was made using HRPWM. (c) Lesion was made with bi-level excitation but cavitation occurred as evidenced by a high number of triggers from the passive cavitation detector.

As in simulation, with the 16 W bi-level excitation, there is a 47% reduction in the mean lesion area which is attributed to the absorption of harmonics. At the two higher powers, however, the reduction in lesion volume is attributed to cavitation. This hypothesis can be confirmed in a number of ways. The average cavitation event count shown in Fig. 5 (purple markers) is high (>10) for the two higher power bi-level exposures, while it is 0 for all other exposures. The lesion data from the 35 W bi-level excitation also failed a normality test that is indicative of the stochastic nature of cavitation. Mechanical damage is further evidenced by comparing the lesion shapes in Fig. 6. Here, all lesions were made using a fixed electrical power of 35 W but with different excitation schemes. The lesion in Fig. 6(a) was made using a linear amplifier and the lesion in Fig. 6(b) was made using HRPWM. These two lesions are axisymmetric and ellipsoid in shape which is desirable. There is no visible evidence of acoustic cavitation and they have a similar cross-section area (a: 53.79 mm^2^, b: 47.29 mm^2^). Lesion (c) was thermally formed using bi-level excitation but cavitation was present causing a change in the absorption. The lesion is round instead of cigar shaped, has a smaller area of 24.34 mm^2^ and the cavitation count reached 255, the maximum possible for the detection scheme used.

**Fig. 7.**
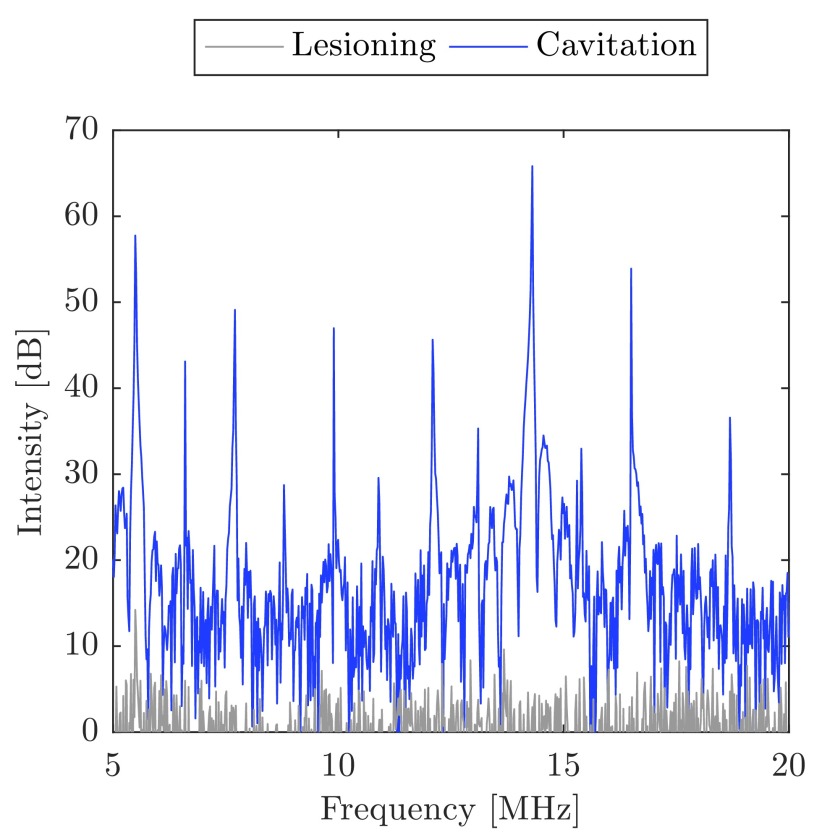
Examples of signals from the passive cavitation detector during lesioning. Gray line: PCD waveform during thermal-only lesioning with the linear amplifier. Blue line: PCD waveform from cavitation during a 26 W bi-level exposure. The reference value used for the intensity calculation is from the mean noise level.

Example signals acquired from the PCD are shown in Fig. 7. The gray line shows an acquired signal during thermal-only lesioning using a 35 W linear amplifier excitation. The spectrum, here, indicates no cavitation activity. The blue line shows an increase in the noise floor and harmonic generation by the presence of bubbles. This cavitation signal was recorded during a 26 W bi-level exposure. The spectrum suggests stable rather than inertial cavitation. At $({21}/{2}) f_{0}$, (11.55 MHz) possible ultraharmonic emissions are visible, which would normally suggest stable cavitation. There are no ultraharmonics visible at the lower end of the spectrum, however, but this may be due to sampling limitations of the PCD or because the exposure pressures were close to the cavitation threshold [73].

### A. Suitability of HRPWM for Therapeutic Ultrasound

**Fig. 8.**
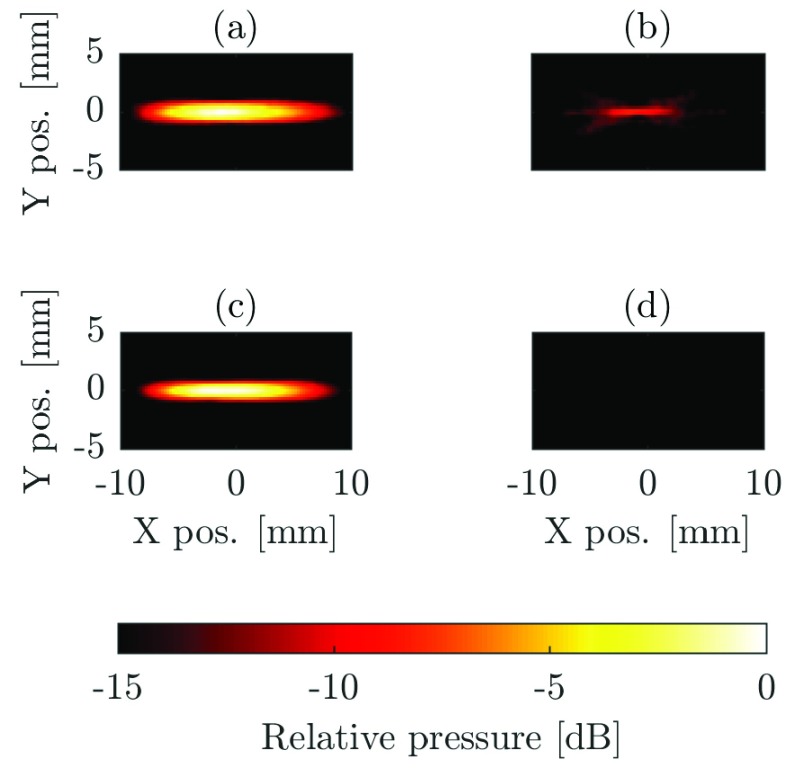
Pressure plots made with (a) bi-level, (c) HRPWM, and (b) third harmonic only with bi-level and (d) HRPWM. For (a) and (c), only the highest 6 dB is shown. For (b) and (d), the highest 15 dB is shown.

Fig. 8 shows the negative pressure beam plots made with the CNC stage and a 0.4 mm membrane hydrophone (D1064, Precicion Acoustics Ltd., Dorchester, U.K.). The step size was 0.5 mm and the peak negative pressure (PNP) was used as an intensity reference.

The acquired waveforms were deconvolved to compensate for the hydrophone response and then an FIR high-pass filter ($n=50$, $f_{c}=2 {\mathrm { \text {M} \text {Hz} }}$) was applied to separate the higher order harmonics from the waveform. Pressure plots in Fig. 8(a) and (c) were produced using the unfiltered waveform of bi-level and HRPWM excitations, respectively. Plots in Fig. 8(b) and (d) show the corresponding plots when the fundamental component is removed. For Fig. 8(a) and (c), only the highest 6 dB is shown. For Fig. 8(b) and (d), the highest 15 dB is shown. The beam plots were made at sufficiently low pressures (<1 MPa) to minimize the harmonic generation by nonlinear propagation. Cubic interpolation was applied to the images so that a 1° rotation could be applied to correct for slight misalignment of the CNC machine and the acoustic path.

These results show that harmonic distortion in the bi-level electrical waveform can be observed in the acoustic domain. HRPWM was effective at reducing harmonic content in the waveform as no harmonics were visible above −15 dB. Since the magnitude of the harmonic components in the bi-level pressure plot and electrical waveform were equal (~−10 dB at 3.3 MHz) this supports the model used in simulation that the transducer is almost equally efficacious at its fundamental component as it is at its third harmonic. This was further confirmed from a bandwidth measurement of the transducer (not shown).

The simulation and experimental parameters at 26 W were very similar although the higher ambient temperature used in the thermal simulations meant that simulated lesions were slightly larger. This was compounded by the fact that the simulation model presumed a 100% efficiency of the transducer, where experimentally only values of 80% have been reported [69]. The simulations showed that an increase in harmonic distortion in the excitation waveform can reduce lesion size for a fixed acoustic power. However, experimentally, a reduction in lesion size with HRPWM was not observed as it was in simulation. This is likely because the transducer is much more effective at attenuating harmonics than the FIR filter used in the simulation model. It is expected that the lesion reduction effect with bi-level excitation would be more pronounced at higher depths as more acoustic energy would be absorbed before the focal region. This is due to bi-level excitation generating harmonics in the acoustic domain which are more readily attenuated. To compensate, larger pressures could be used, but this is undesirable as it increases the likelihood of cavitation.

Experimentally, additional undesirable mechanical effects were observed when using bi-level excitation. This was evidenced by the PCD data, observations from the shape of the lesion, and from statistical tests. The likely nonnormality of the 35 W results may have influenced the ANOVA, but this is unlikely [74]. The authors believe the cause of cavitation was the result of waveform shape rather than any inherent harmonic distortion. This suggests that a regular five-level staircase circuit might suffice, although to the best of the authors knowledge no other appropriate PWM algorithm exists. Transient response is often related to applied frequency, so further work should involve investigating whether the applied frequency influences the nuclearion of cavitation.

HRPWM’s major advantage over other schemes, however, is its ability to apodise an array without the need for each element to have an independent power supply or filter. This makes it suitable for a number of high-density applications, catheters, and MRI environments, especially if combined with new techniques to replace the matching network [75]. Although it was necessary to design an amplifier for this study, the algorithm could also be targeted to other circuit designs if they allow. The experimental results show that this switching algorithm and circuit design can control thermal dose at intensity levels useful in HIFU.

### B. Cavitation Nucleation With Bi-level Excitation

It was not possible to generate thermal-only lesions using 35 W bi-level excitation. Cavitation activity was also observed during two of the three exposures at 26 W using bi-level excitation. To discount possible error in power measurement, the acoustic intensity of the bi-level and linear amplifier schemes was compared at each power level. The −6 dB beamwidth was measured as 1.85 mm using the membrane hydrophone and the time-averaged acoustic power for each electrical power was measured with a radiation force balance (Precision Acoustics Ltd., Dorchester, U.K.) [76]. Due to the harmonic components having different focal positions, bi-level excitation distorted the focal region slightly, but it did not have any significance at the −6 dB threshold. At each power level, the intensities were found to be similar irrespective of the excitation scheme used. This means that the cavitation with bi-level excitation was not nucleated primarily through heating.

Experiments were repeated in an agar phantom to compare with chicken breast and it was found that cavitation was also observed using the 26 W bi-level excitation, but not using the linear amplifier, even at higher intensities. The first cavitation event was temporally located at $85~\mu \text{s}$ after the start of firing, which is approximately twice the propagation time for the natural focus point of the transducer. This suggests that a transient near the start of the waveform was nucleating the cavitation.

**Fig. 9.**
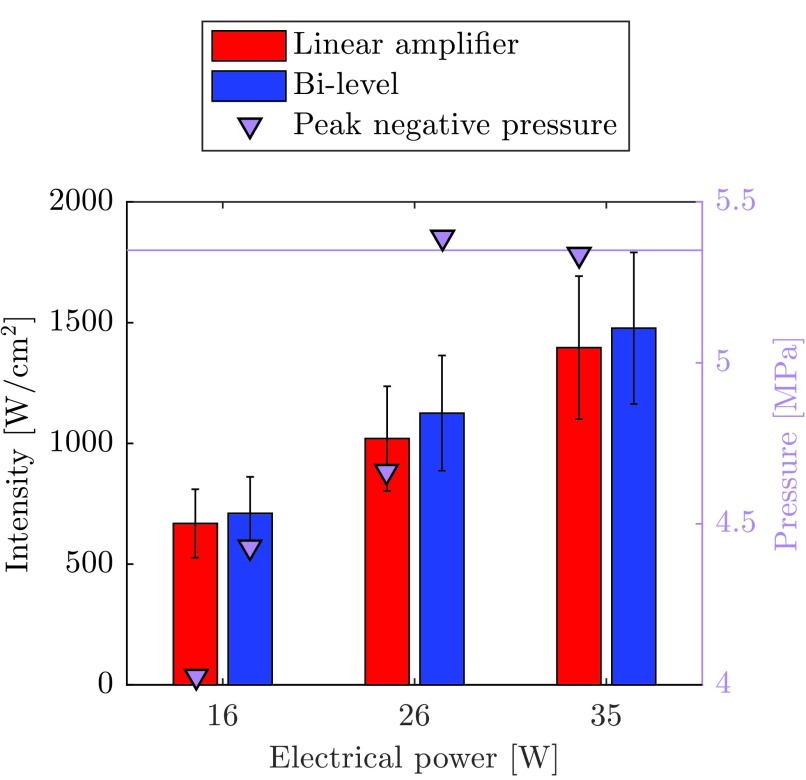
Measured acoustic intensity and transient pressure values for linear amplifier and bi-level excitations at all three electrical powers. Due to saturation of the hydrophone, it was not possible to obtain pressure measurements for bi-level at 35 W. Pink line: suspected cavitation threshold.

Subsequent high resolution ($f_{s}$ = 200 MHz) acoustic waveforms were captured using the membrane hydrophone at the focal point. It was observed that the pressure when using the linear amplifier increased slowly over the first three cycles but when using bi-level excitation, there were large negative transients. Because of the saturation limit of the hydrophone, it was not possible to observe these transients with the highest power bi-level excitation. The values of these transients are shown alongside intensity measurements in Fig. 9. The error bars represent minimum and maximum values of intensity caused by spatial averaging of the hydrophone and the purple line represent the suspected cavitation threshold. By virtue of the measured acoustic powers being similar, the results also show that the electrical power measurement technique was adequate for comparing each scheme.

A high PNP makes cavitation more likely, and so cavitation nucleation could be attributed to the negative pressure transients. With 26 W bi-level excitation, the PNP was larger than the PNP with the linear amplifier at both 26 and 35 W power levels. It is also expected that these transients are very close to the cavitation threshold as it was also not possible to produce thermal-only lesions with the linear amplifier at 38 W.

The cause for cavitation with bi-level excitation is not fully understood and warrants further investigation. It likely involves a number of factors including transients preconditioning the tissue [77] and focal region distortion which have both been observed and could be exacerbated by nonlinear propagation in tissue.

## VI. Conclusion

HRPWM was compared with a linear amplifier and bi-level excitations numerically and experimentally. In simulation, it was shown that increased harmonic distortion in the bi-level excitation waveform reduced the size of lesions. For the experiment, three waveforms of different powers were generated by adjusting the modulation parameter of HRPWM. Using these waveforms, lesions in *ex vivo* tissue were made and compared against lesions made at equivalent electrical powers using bi-level excitation and a linear amplifier. The experimental results showed that HRPWM could produce thermal-only lesions of an equivalent size to those made with a linear amplifier. At the lowest power-level used, bi-level excitation produced smaller lesions, but transients in the waveform nucleated cavitation at the higher power levels.

The study showed that HRPWM was able to control acoustic pressure at intensities relevant to HIFU. The use of HRPWM will facilitate improvements in efficiency, practicality, and cost for HIFU drive circuitry.
